# P28 ESR – master of disguise in a case of rheumatoid arthritis

**DOI:** 10.1093/rap/rkad070.049

**Published:** 2023-09-27

**Authors:** Rabia Gull, Geetha Lakshmi Janakiraman

**Affiliations:** James Cook University Hospital, South Tees NHS Foundation Trust, Middlesbrough, United Kingdom; James Cook University Hospital, South Tees NHS Foundation Trust, Middlesbrough, United Kingdom

## Abstract

**Introduction:**

ESR is one of the inflammatory markers widely used by rheumatologists. Though ESR is a simple, less expensive test, it lacks sensitivity and specificity. When it is significantly elevated it always raises the suspicion of infection/inflammation/malignancy; further investigation to look for the cause of elevation is paramount. The current case highlights the importance of a broad differential diagnosis when investigating raised inflammatory markers and correlating them clinically. The raised ESR can mask the potentially serious diagnosis of malignancy overlapping with the autoimmune process.

**Case description:**

A 66-year-old gentleman presented with 4 months' history of arthralgia and joint stiffness especially in the hands and feet. He had a preceding COVID-19 illness. He was hypertensive on ramipril. He spoke minimal English and was mostly accompanied by his family members who translated. He was an ex-smoker who was independent and worked in a shop. He exhibited a high DAS 28 score of 7.61 on a background of strongly positive CCP >340 and rheumatoid factor 79.8. His inflammatory markers were raised with ESR 52 and CRP 17.

He was started on methotrexate oral weekly maintenance. His folic acid was increased due to nausea. At 2 months' review due to persistent high disease activity with DAS28, 5.74 sulfasalazine was added. Due to nausea and vomiting on higher doses of sulfasalazine, the dose was reduced and subsequently stopped by the patient. Methotrexate was switched to sub-cutaneous injections.

At 6 months' review he reported persistent vomiting and some weight loss with rising inflammatory markers ESR 125/CRP 65. Due to the persistent active RA, he was referred to start tocilizumab while an urgent CT chest, abdomen, and pelvis (CT CAP) was requested. In the interim, he was admitted with headaches and a general decline. The CT CAP was delayed due to the COVID-19 pandemic but revealed primary lung malignancy with widespread metastasis including the brain, supra, and infra diaphragmatic spread.

Meanwhile, his general health and functional status rapidly declined. He was readmitted with hemoptysis, progressive weight loss, and shortness of breath. The lung MDT decision was the best supportive care. Sadly, he passed away soon after.

**Discussion:**

This patient had presented with difficult-to-control seropositive RA during the COVID-19 pandemic post-COVID infection. These were challenging times with restricted face-to-face appointments and delays in diagnostics. There was limited to no clinical examination and reliance on the patient’s description of clinical presentation. English was not the first language of the patient and communication might not have been effective in describing the symptoms. The face-to-face reviews with clinical examination might have helped in the early investigation and timely diagnosis and treatment of the malignancy.

Even though the persistently raised inflammatory markers raised suspicions regarding underlying malignancy enough to order an urgent CT CAP, there was over 4 weeks' delay in getting this done. His poorly responding RA was attributed to intolerance to the conventional disease-modifying anti-rheumatic drugs (DMARDs) and persistent vomiting resulting in poor abortion of DMARDs.

This was an unfortunate case in which the adverse outcome might have been avoided or delayed by early diagnosis and treatment of the underlying malignancy.

**Key learning points:**

Keep an open mind for a differential diagnosis.

ESR is a nonspecific investigation and should be interpreted with caution to include wider differentials including malignancy.

Telephone consultations are a great asset for the right patients but certainly, face-to-face appointments for appropriate cases will help clinicians to perform thorough clinical examination and prompt investigation and treatments.

Increased vigilance is essential to escalate/re-evaluate differentials when there is a poor response to treatment.

Importance of face-to-face consultations and thorough clinical examination.

